# Microablative fractional radiofrequency for sexual dysfunction and vaginal Trophism: A randomized clinical trial

**DOI:** 10.1016/j.clinsp.2023.100293

**Published:** 2023-10-13

**Authors:** Ayane Cristine Alves Sarmento, Fabíola Sephora Fernandes, Rafaella Rêgo Maia, Juliana Dantas de Araújo Santos Camargo, Janaina Cristiana de Oliveira Crispim, José Eleutério Júnior, Ana Kataherine Gonçalves

**Affiliations:** a Health Sciences Postgraduate Program, Universidade Federal do Rio Grande do Norte (UFRN), Natal, RN, Brazil; b Department of Clinical Analysis and Toxicology, Universidade Federal do Rio Grande do Norte (UFRN), Natal, Brazil; c Departamento Obstetrics and Gynaecology, Universidade Federal do Ceara, Ceara, CE, Brazil; d Department of Obstetrics and Gynaecology, Universidade Federal do Rio Grande do Norte (UFRN), Natal, RN, Brazil

**Keywords:** Menopause, Atrophy, Sexual dysfunction, Radiofrequency Therapy

## Abstract

**Objectives:**

To evaluate Microablative Fractional Radiofrequency (MAFRF) as a possible option in treating vaginal atrophy**.**

**Methods:**

This was a randomized, controlled clinical trial with postmenopausal women diagnosed with vaginal atrophy. The treatment consisted of three sessions of MAFRF, compared to vaginal estrogen administration and an untreated control group. Assessments occurred at baseline and 90 days. The primary endpoints were sexual function, evaluated by the Female Sexual Function Index (FSFI), and vaginal health, assessed by the Vaginal Health Index (VHI). Secondary outcomes included vaginal microbiota composition (Nugent score) and epithelial cell maturation (Maturation Value ‒ MV).

**Results:**

One hundred and twenty women (40 in each group) were included. Concerning the FSFI, both groups, MAFRF (median 4.8 [3.6‒6.0]) and vaginal estrogen (mean 4.7 ± 1.1), experienced improved sexual desire when compared to the control group (median 3.6 [2.4‒4.8]). Regarding the total score of VHI, the authors observed an improvement in the mean of the MAFRF (23.7 ± 2.0) and vaginal estrogen groups (23.5 ± 1.9) when compared to the control (14.8 ± 2.9). The Nugent score was reduced in the MAFRF and estrogen groups (p < 0.01) compared to the control group. Lastly, the MV was modified after treatment with MAFRF (p < 0.01) and vaginal estrogen (p < 0.001). No differences existed between the MAFRF and vaginal estrogen groups in the studied variables. No adverse effects were reported following the MAFRF protocol.

**Conclusions:**

Radiofrequency was comparable in efficacy to estrogen administration for treating vulvovaginal atrophy. It deserves consideration as a viable option in managing this condition.

## Highlights

• No significative adverse effects were reported following the Radiofrequency protocol.
• Radiofrequency was comparable in efficacy to estrogen for vulvovaginal atrophy.
• Radiofrequency could be a viable option in vulvovaginal atrophy management.

## Introduction

During menopause, estrogen deficiency induces atrophic changes in the urogenital tract epithelium, resulting in vaginal discomfort, dryness, burning, irritation/pain, and this can be accompanied by an increased occurrence of sexual and urinary discomfort and urinary tract infections.^1^^,^^2^ In addition, there are significant modifications in the composition of the vaginal microbiota. Due to a decrease in estrogen levels, there is a reduction in glycogen deposition on vaginal epithelial cells. This results in a decrease in *Lactobacillus spp*., an increase in Gram-negative bacteria, and an elevation in vaginal pH. The decline in estrogen also induces a decrease in maturation of the squamous epithelium, with a consequent increase in basal and intermediate cells and a reduction in superficial cells.^2^^,^^3^ Decrease in vaginal elasticity, increased roughness, and the presence of petechiae are significant consequences of low epithelial maturation.^1^, ^2^, ^3^, ^4^

Hypoestrogenism also provokes alteration in collagen type I fibrils to collagen type III fibrils ratio with loss of their trabecular disposition, decreased quantity of elastic fibers, reduced vascularization, and thinning and flattening of the vaginal epithelium, which can superficially turn into a keratinized layer.^5^^,^^6^

Several strategies have been proposed to improve the symptoms of menopause. Hormonal (estrogens and androgens) and non-hormonal (lubricants and long-acting vaginal moisturizers) therapies are most frequently employed. Vaginal estrogen is the acknowledged gold standard for the treatment of urogenital atrophy. However, non-hormonal approaches can be beneficial in cases where hormonal therapy is contraindicated, such as in women with hormone-dependent cancers (breast/endometrial cancer) or when the woman chooses to avoid hormonal exposure.^1^^,^^3^^,^^7^^,^^8^

The energy-based devices in gynecology have been used in the last years; the most common are laser and radiofrequency (RF). The CO_2_ Laser is authorized by the Food and Drug Administration (FDA) for general indications of gynecological instruments, including the destruction of abnormal cervical or vaginal tissue, condylomata, and precancerous lesions.^9^ In 2020, the North American Menopause Society issued a norm regarding the use of energy-based devices in the Genitourinary Syndrome of Menopause (GSM) due to the several clinical studies that have shown them to be effective in treating the syndrome.^4^, ^5^, ^6^, ^7^, ^8^, ^9^, ^10^, ^11^

The thermal effects of radiofrequency induce collagen denaturation, promoting the immediate and effective contraction of its fibers, activating fibroblasts, and leading to neocolagenesis, the reorganization of collagen fibers, and subsequent tissue remodeling.^8^ Micro-Ablative Fractional Radiofrequency (MAFRF) is a new procedure that uses random energy in a fractionation system that provokes thermal relaxation of the tissue at a specific time. The fractioned energy is distributed at equidistant points, producing microscopic columns of thermal lesions in the epidermis and upper dermis, resulting in microscopic columns of treated tissue interspersed with areas of untreated skin allowing for faster re-epithelialization.^8^, ^9^, ^10^, ^11^ Based on the new concept of mucosa remodeling using optical microscopy patterns of thermal damage caused by physical energies, this study aims to evaluate using the MAFRF as a possible option in treating vaginal atrophy.

## Methods

The authors conducted a randomized controlled clinical trial to compare the therapeutic responses to MAFRF treatment with that of vaginal estrogen (Estradiol 3-propyl17β-methyldiether-based) and untreated controls. This study was conducted at a gynecological unit of a public university hospital between July 2020 and September 2022.

Participants were selected via referrals from gynecologist physicians at the Januário Cicco Maternity/Federal University of Rio Grande do Norte. The Onofre Lopes Hospital Research Ethics Committee/ Federal University of Rio Grande do Norte approved the trial (81973618.2.0000.5292) and was registered in the Clinical Trials Registry (ReBec) ‒ (RBR-94DX93) https://ensaiosclinicos.gov.br/rg/RBR-94dx93. Written informed consent was obtained from all subjects. Consolidated Standards of Reporting Trials (CONSORT) guidelines were followed.^12^

Participants were eligible if they were healthy postmenopausal women (55 to 65 years old, with at least 12 months elapsed since their last menstrual period or bilateral oophorectomy), sexually active (at least one vaginal sexual intercourse per week with a partner), with vulvovaginal atrophy (VHI ≤ 15), plasma gonadotropin, and serum hormonal levels in the postmenopausal range (FSH > 40 mIU/mL; estradiol < 25 pg/mL). All women were sexually active and had a normal Pap test and a negative HPV test.

**Fig. 1:**
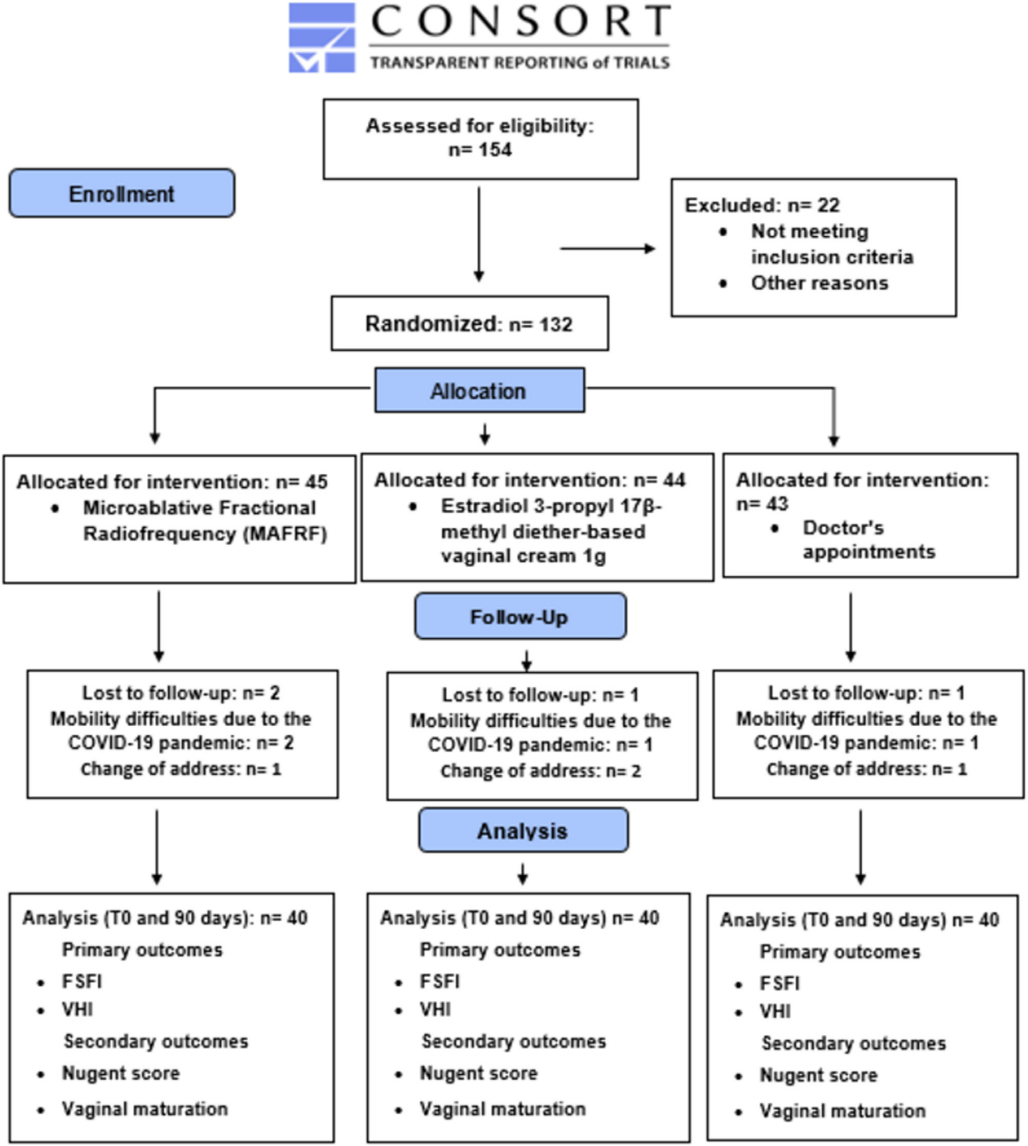
CONSORT 2010 Flow Diagram.

Exclusion criteria were patients who used any form of hormonal (systemic or local) therapy in the last six months, lubricants, or vaginal moisturizers in the previous month, with active genital infections tested by real-time multiplex PCR (AnyplexTM II STI-7 Detection). The authors excluded patients with vulvar dermatological disorders, vulvar squamous cell carcinoma precursors, lower anogenital squamous lesions, and vulvodynia. The study flowchart is shown in Fig. 1.

Participants were randomized into one of three treatment arms: MAFRF; Vaginal estrogen: Estradiol 3-propyl 17β-methyldiether; Controls: untreated.

The participants were randomized to one of the three arms in a 1:1:1 ratio based on computer-generated randomization (Software Research Randomizer® program). To ensure allocation concealment, an offsite randomization schedule was used. The randomization schedule was prepared in advance by a researcher, who had no contact with any participants throughout the trial and had not been involved in the recruitment, screening, assessment, enrollment, or treatment process. To enroll a participant, the primary researcher sent an e-mail containing the consenting participant's name to the researcher. These details were entered into the allocation spreadsheet, and the subsequent treatment allocation and participant identification number were emailed directly to the treatment.

The MAFRF was performed according to the technique described by Kamilos and Borelli.^7^ For the procedure, the Wavetronic 6000 Touch device was used with the Megapulse HF FRAXX system (Loktal Medical Electronics), equipped with an electronic circuit of energy fractionation, connected to a vaginal pen with 64 microneedles, 200 μ in diameter and 1 mm in length, mounted on a Teflon body and divided into an eight-column matrix with eight needles each.^7^

In the vestibule and vaginal opening, 10% lidocaine spray was applied 3 minutes before the procedure. Three applications were performed in the vagina/vaginal introitus, at intervals of 30 days. A sequential application was performed on the vaginal walls under direct visualization. For post-treatment care, a 5% dexpanthenol solution was applied to the vaginal opening twice a day for 2 to 5 days. Subjects were told to avoid sexual intercourse for ten days.^7^ The procedure was performed in the outpatient clinic by an experienced gynecologist, and a single gynecologist supervised the process for the entire research period.

Patients in the estrogen group were instructed to use I gm of Estradiol 3-propyl 17β-methyl diether-based vaginal cream, twice a week, for three months.^3^^,^^13^

Women in the control group received physician appointments during the follow-up period, where they received clarification about the GSM and guidelines about the principal conducts for managing symptoms provoked by these conditions.

At their initial visit, the women answered a standardized questionnaire with baseline demographic data, including age, Body Mass Index (BMI), parity, years since menopause, and prior use of any hormonal therapy. Two relevant time points were considered for evaluating treatment results (days 0 and 90).

The primary outcomes were sexual function, evaluated by the Female Sexual Function Index (FSFI), and vaginal health, assessed by the Vaginal Health Index (VHI). Secondary outcomes included composition of the vaginal microbiota (Nugent score) and Vaginal Maturation (MV).

The FSFI is a brief scale for assessing female sexual function. It is a written test with six subscales and one sum of scores that measures the degree of desire/excitement/lubrication/orgasm/satisfaction, and pain. The subscales' scores are correlated and added, resulting in a final score. Final scores can range from 2 to 36, and higher scores indicate a better degree of sexual function.^14^^,^^15^

The vaginal health score consists of a clinical analysis performed during a speculum-based examination. It measures five parameters (elasticity, fluid volume, pH, epithelial cell integrity and humidity), graded from 1 to 5. The sum of the parameters evaluated results in the total vaginal health score. When the overall rating is less than 15, the vaginal mucosa is considered atrophic.^2^^,^^4^^,^^16^ For vaginal pH determination, the pH indicator strips 4.0‒7.0 (MColorpHastTM, Merck, Germany) were applied against the vaginal wall.

A sample was obtained from the right side wall of the vaginal canal. The swab contents were transferred to a slide and stained by the Gram method. The bacterial morphotypes that were visualized were quantified using Nugent's score. This method classifies bacteria into *Lactobacillus* morphotypes (long Gram-positive bacilli), *Gardnerella spp*. and *Bacteroides spp*. (Gram-negative or Gram variable *coccobacilli*), and *Mobiluncus spp.* (Gram-negative curved bacilli). Each morphotype is quantified, and according to the score obtained, the vaginal sample is classified as normal (0‒3), intermediate (4‒6), or bacterial vaginosis (7‒10).^17^^,^^18^

Vaginal smear samples were collected from the upper distal third of the right lateral wall using an Ayre spatula. Parabasal (P), Intermediate (I), and Superficial (S) cell counts were obtained to determine the degree of atrophy based on the Frost Maturation Index (MI). The MI was then used to calculate the maturation value (MV = [0×%P] + [0.5×%I] + [1×%S]). MV values ranging from 0 to 49 indicate a low estrogen effect, 50 to 64 indicate a moderate estrogen effect, and 65 to 100 indicate a high estrogen effect on the vaginal epithelium. All samples were examined by an experienced cytopathologist blinded to the treatment regimens.^2^^,^^19^

All possible adverse effects were recorded and qualified using questionnaires developed for this protocol during treatment. Any breaches of confidentiality, study protocol, or Adverse Events (AEs) attributable to this study were reported to the research ethics committees. The researchers who analyzed the study data were unaware of the treatment applied to any given group.

The sample calculation was performed from the effect size of 0.43 obtained from the FSFI score variation.^7^ Considering an 80% power and alpha error of 5%, the sample size was 36. Due to possible losses during the sampling, the authors added 20% to the calculated value, totaling 43 participants in each group.

The Shapiro-Wilk normality test was applied to verify the adherence of continuous variables to the normal distribution. The descriptive analysis of the variables that adhered to the normal distribution was performed using the mean and standard deviation (Mean ± SD). For variables that did not present a normal distribution in any of the groups, the median and the 25^th^ and 75^th^ percentiles were used. The analysis was performed using absolute and relative frequencies for categorical variables. The Chi-Square test was used to analyze the association between categorical variables. The paired-samples *t*-test was performed to determine whether there were differences between the scores at the two evaluated times (0 and 90 days). Inspection of outliers (box-plot) and normality (Shapiro-Wilk test) was carried out in the distribution of differences between times. In the presence of outliers, the non-parametric Wilcoxon test was used. For results that showed significance in the *t*-test, Cohen's d effect size was calculated by dividing the mean difference by the standard deviation of the difference. For the Wilcoxon test, the effect size r was calculated according to the equation r = z/√n. Values were evaluated according to the Cohen scale: up to 0.20 = small; between 0.20 and 0.79 = moderate and ≥0.80 = major (Cohen, 1988). One-way anova and Tukey's post hoc tests were performed to assess differences within groups in each of the study variables at both time points (before and at 90 days). The assumptions for carrying out the tests were evaluated by inspecting the box-plot (outliers), the Shapiro-Wilk test (normality) and the Levene test (homogeneity of variances). In cases where outliers were detected, the Kruskal-Wallis test was used and, subsequently, pairwise comparisons were performed using the Dunn procedure (1964) with a Bonferroni correction for multiple comparisons. In the presence of heterogeneity of variances, Welch's Anova and Games-Howell's post hoc test were used. A significance level of 5% was adopted for all analyses. All analyses were performed using the software SPSS version 28.0 (Statistical Package for the Social Sciences, Chicago, EUA).

## Results

A total of 120 women were included in the survey showing the following characteristics: median (range) age: 59 (55‒65), years of menopause: 7 (5‒10), body mass index (kg/m^2^), 25.6 (24.7‒26.9), number of pregnancies: 2 (2‒3), smoker: 29.2% and previous hormone therapy: 37.5%. No differences were observed between the three groups (p>0.05) regarding age, time since menopause, body mass index, number of pregnancies, smoking status, and previous hormone therapy (Table 1).

**Table 1:** Characterization of participants by treatment group.

Variables	n (%)	Group			p-value^a^
		MAFRF (n = 40)	Vaginal estrogen (n = 40)	Control (n = 40)	
Age, years	120	57 (55‒62)	59 (55‒65)	61 (55‒65)	0.281
Menopause, years	120	7 (5‒10)	7 (5‒10)	8 (5‒10)	0.789
Body mass index, kg/m^2^	120	25.8 ± 1.6	25.8 ± 1.5	25.5 ± 1.4	0.533
No. pregnancies	120	2 (1‒3)	2 (2‒3)	2 (2‒3)	0.325
Smoker, n (%)					0.754
Yes	35 (29.2)	10 (25.0)	12 (30.0)	13 (32.5)	
No	85 (70.8)	30 (75.0)	28 (70.0)	27 (67.5)	
Previous hormone therapy, n (%)					0.726
Yes	45 (37.5)	14 (35.0)	14 (35.0)	17 (42.5)	
No	75 (62.5)	26 (65.0)	26 (65.0)	23 (57.5)	

a Significance of the difference between groups by ANOVA or Kruskal-Wallis test (continuous variables) or Pearson's Chi-Square test (categorical variables). Continuous data are expressed as mean and standard deviation: ME ± SD or as median and 25^th^ and 75^th^ percentiles: MED (P25‒P75). Categorical data are expressed in absolute (n) and relative (%) frequencies. MAFRF, Microablative Fractional Radiofrequency; kg, Kilograms; m, meters.

Regarding the FSFI scores, there was no difference at baseline in the total FSFI score, thus ensuring homogeneity among the three groups. Both groups, MAFRF (median 4.8 [3.6‒6.0]) and vaginal estrogen (mean 4.7±1.1), experienced improved sexual desire when compared to the control group (median 3.6 [2.4‒4.8]) p = 0.005. Regarding the total FSFI score, the authors observed an improvement before and after treatment for the MAFRF group (median [interquartile range]: 14.2 [12.4‒17.8] - 17.4 [14.4‒22.3] p < 0.01, however, no difference was observed between groups after 90 days, p = 0.557, Table 2).

**Table 2:** Comparison of FSFI scores before and after treatment.

Variables	Groups	Time		p-value^a^	Multiple comparisons	Post hoc^c^	
		Baseline	90 days			Baseline	90 days
Desire	MAFRF	3.6 (2.4‒5.4)	4.8 (3.6‒6.0)	**0.008**	MAFRF vs. Vaginal estrogen	0.065	0.971
	Vaginal estrogen	4.6 ± 1.1	4.7 ± 1.1	0.609	MAFRF vs. Control	0.170	**0.017**
	Control	4.8 (3.6‒6.0)	3.6 (2.4‒4.8)	**0.005**	Vaginal estrogen vs. Control	0.962	**0.006**
p-value^b^		0.072	**0.005**				
Excitement	MAFRF	1.8 (0.0‒3.2)	3.3 (1.5‒4.8)	**p < 0.01**	MAFRF vs. Vaginal estrogen	0.227	0.325
	Vaginal estrogen	3.0 (1.5‒3.8)	2.6 (1.5‒4.1)	0.142	MAFRF vs. Control	0.350	0.559
	Control	2.7 (1.2‒3.3)	2.7 (1.5‒3.6)	0.437	Vaginal estrogen vs. Control	0.962	0.914
p-value^b^		0.210	0.337				
Lubrication	MAFRF	2.1 (0.0‒3.6)	3.6 (0.0‒3.9)	**0.006**	MAFRF vs. Vaginal estrogen	0.668	0.962
	Vaginal estrogen	3.0 (0.0‒3.6)	3.3 (0.0‒3.6)	0.079	MAFRF vs. Control	0.239	0.987
	Control	3.2 (0.0‒3.9)	3.3 (0.5‒3.8)	0.874	Vaginal estrogen vs. Control	0.723	0.909
p-value^b^		0.270	0.915				
Orgasm	MAFRF	2.0 (0.0‒3.6)	3.6 (0.0‒4.0)	**p < 0.01**	MAFRF vs. Vaginal estrogen	0.396	0.930
	Vaginal estrogen	2.8 (0.0‒4.0)	3.2 (0.0‒4.0)	**0.043**	MAFRF vs. Control	0.272	0.659
	Control	2.8 (0,0‒4,0)	2.8 (0.0‒3.6)	0.793	Vaginal estrogen vs. Control	0.967	0.867
p-value^b^		0.254	0.682				
Satisfaction	MAFRF	2.4 (2.1‒2.4)	2.4 (2.0‒3.5)	0.247	MAFRF vs. Vaginal estrogen	‒*	‒*
	Vaginal estrogen	2.4 (2.0‒2.4)	2.4 (2.0‒3.1)	0.597	MAFRF vs. Control	‒*	‒*
	Control	2.4 (2.0‒3.2)	2.4 (2.0‒3.2)	0.414	Vaginal estrogen vs. Control	‒*	‒*
p-value^b^		0.643	0.539				
Pain	MAFRF	3.9 ± 2.3	3.2 ± 2.3	0.088	MAFRF vs. Vaginal estrogen	0.424	0.904
	Vaginal estrogen	2.8 (1.6‒6.0)	2.8 (1.6‒6.0)	0.113	MAFRF vs. Control	0.800	0.706
	Control	4.0 (2.4‒5.7)	4.0 (1.4‒5.1)	0.597	Vaginal estrogen vs. Control	0.811	0.928
p-value^b^		0.458	0.729				
Total	MAFRF	14.2 (12.4‒17.8)	17.4 (14.4‒22.3)	**p < 0.01**	MAFRF vs. Vaginal estrogen	0.307	0.723
	Vaginal estrogen	16.8 (14.5‒20.6)	17.0 (13.3‒20.9)	0.069	MAFRF vs. Control	0.086	0.549
	Control	16.1 (14,0‒22,0)	14.7 (13.8‒21.6)	0.335	Vaginal estrogen vs. Control	0.782	0.958
p-value^b^		0.095	0.557				

a Significance of the difference between the treatments (paired *t*-test or Wilcoxon test).

b Significance of the difference between the groups (One-way Anova or Welch's Anova or Kruskal-Wallis).

c Significance of the comparisons between groups (Tukey or Games-Howell or Dun/Benferroni tests). Continuous data are expressed as mean and standard deviation: ME ± SD or as median and 25^th^ and 75^th^ percentiles: MED (P25‒P75). Values in bold indicate significance at p < 0.05.

⁎ It is not possible to run post hoc testing for non-significant results in the Kruskal-Wallis test. FSFI, Female Sexual Function Index; MAFRF, Microablative Fractional Radiofrequency.

Concerning vaginal health, there was no difference at baseline in the total VHI score, thus ensuring homogeneity between groups. The authors found statistical significance to vaginal humidity, fluid volume, pH, elasticity, and integrity (p < 0.01) of the MAFRF and vaginal groups. In the total score of VHI, the authors observed an improvement in the mean of the MAFRF (mean [23.7 ± 2.0]) and vaginal estrogen groups (mean [23.5 ± 1.9]) when compared to the control (mean [14.8 ± 2.9]) p < 0.01 (Table 3).

**Table 3:** Comparison of VHI scores before and after treatment.

Variables	Groups	Time		p-value^a^	Multiple comparisons	Post hoc^c^	
		Baseline	90 days			Baseline	90 days
Vaginal humidity	MAFRF	2.2 ± 0.4	4.8 ± 0.4	**p < 0.01**	MAFRF vs. Vaginal estrogen	‒*	1.000
	Vaginal estrogen	2.3 ± 0.5	4.8 ± 0.4	**p < 0.01**	MAFRF vs. Control	‒*	**p < 0.01**
	Control	2.1 ± 0.3	3.2 ± 0.7	**p < 0.01**	Vaginal estrogen vs. Control	‒*	**p < 0.01**
p-value^b^		0.144	**p < 0.01**				
Fluid volum	MAFRF	2.0 (2.0‒3.0)	5.0 (4.3‒5.0)	**p < 0.01**	MAFRF vs. Vaginal estrogen	0.973	0.536
	Vaginal estrogen	2.0 (2.0‒3.0)	5.0 (4.0‒5.0)	**p < 0.01**	MAFRF vs. Control	1.000	**p < 0.01**
	Control	2.4 ± 0.5	2.8 ± 0.6	**0.003**	Vaginal estrogen vs. Control	0.973	**p < 0.01**
p-value^b^		0.967	**p < 0.01**				
pH	MAFRF	3.2 ± 1.6	4.8 ± 0.7	**p < 0.01**	MAFRF vs. Vaginal estrogen	‒*	0.616
	Vaginal estrogen	4.0 (3.0‒4,0)	5.0 (5.0‒5.0)	**p < 0.01**	MAFRF vs. Control	‒*	**p < 0.01**
	Control	3.0 (1.0‒4.0)	3.0 (1.0‒4.0)	0.782	Vaginal estrogen vs. Control	‒*	**p < 0.01**
p-value^b^		0.179	**p < 0.01**				
Elasticity	MAFRF	2.3 ± 0.5	4.8 ± 0.4	**p < 0.01**	MAFRF vs. Vaginal estrogen	1.000	0.263
	Vaginal estrogen	2.3 ± 0.5	4.6 ± 0.5	**p < 0.01**	MAFRF vs. Control	0.968	**p < 0.01**
	Control	2.3 ± 0.5	3.1 ± 0.8	**p < 0.01**	Vaginal estrogen vs. Control	0.968	**p < 0.01**
p-value^b^		0.961	**p < 0,01**				
Integrity	MAFRF	3.0 (2.0-3.0)	5.0 (4.0-5.0)	**p < 0.01**	MAFRF vs. Vaginal estrogen	0.440	0.457
	Vaginal estrogen	2.7 ± 0.5	4.5 ± 0.7	**p < 0.01**	MAFRF vs. Control	**0.002**	**p < 0.01**
	Control	3.0 (3.0‒3.0)	3.0 (3.0‒3.0)	0.467	Vaginal estrogen vs. Control	**0.021**	**p < 0.01**
p-value^b^		**0.006**	**p < 0.01**				
Total	MAFRF	12.8 ± 1.8	23.7 ± 2.0	**p < 0.01**	MAFRF vs. Vaginal estrogen	0.474	0.914
	Vaginal estrogen	13.2 ± 1.5	23.5 ± 1.9	**p < 0.01**	MAFRF vs. Control	0.813	**p < 0.01**
	Control	12.5 ± 1.9	14.8 ± 2.9	**p < 0.01**	Vaginal estrogen vs. Control	0.181	**p < 0.01**
p-value^b^		0.187	**p < 0.01**				

a Significance of the difference between the moments (paired *t*-test or Wilcoxon test).

b Significance of the difference between groups (One-way Anova or Welch's Anova or Kruskal-Wallis).

c Comparisons between groups (Tukey or Games/Dunnferroni test). Continuous data are expressed as mean and standard deviation: ME ± SD or as median and 25^th^ and 75^th^ percentiles: MED (P25-P75). Values in bold indicate significance at p<0.05.

⁎ It is not possible to run the post hoc test for results not achieved in the Kruskal-Wallis test. VHI, Vaginal Health Index; MAFRF, Microablative Fractional Radiofrequency.

**Fig. 2:**
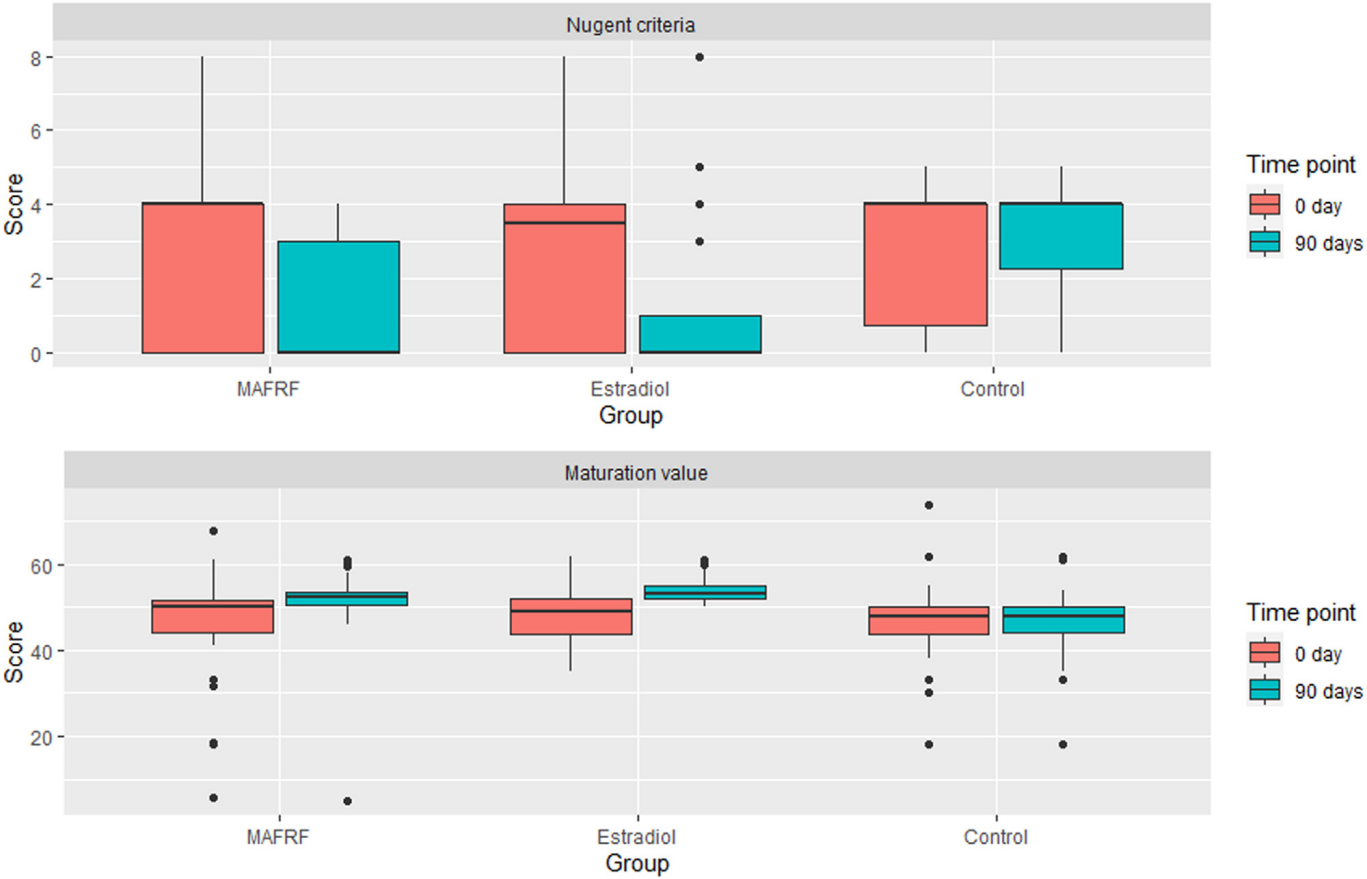
Comparison of total Nugent score and MV before and after treatment.

There was a decrease of 4.0 points in the Nugent scores in the MAFRF group between baseline (4.0) and after 90 days (0.0), *z* = 3.676, p < 0.01, *r* = -0.58, indicating a moderate change. There was also a reduction in Nugent scores in the vaginal estrogen group of 3.5 points between baseline (3.5) and after 90 days (0.0), *z* = -2.958, p = 0.003, *r* = -0.47, indicating a similar effect (Fig. 2). There were differences between the three groups in the Nugent score after 90 days (p < 0.01). Post hoc analysis revealed differences in median scores between MAFRF (0.00) and Control (4.00) groups (p < 0.01) and between vaginal estrogen (0.00) and Control (4.00) groups (p < 0.01), but not between MAFRF and vaginal estrogen (p = 0.552).

**Fig. 3:**
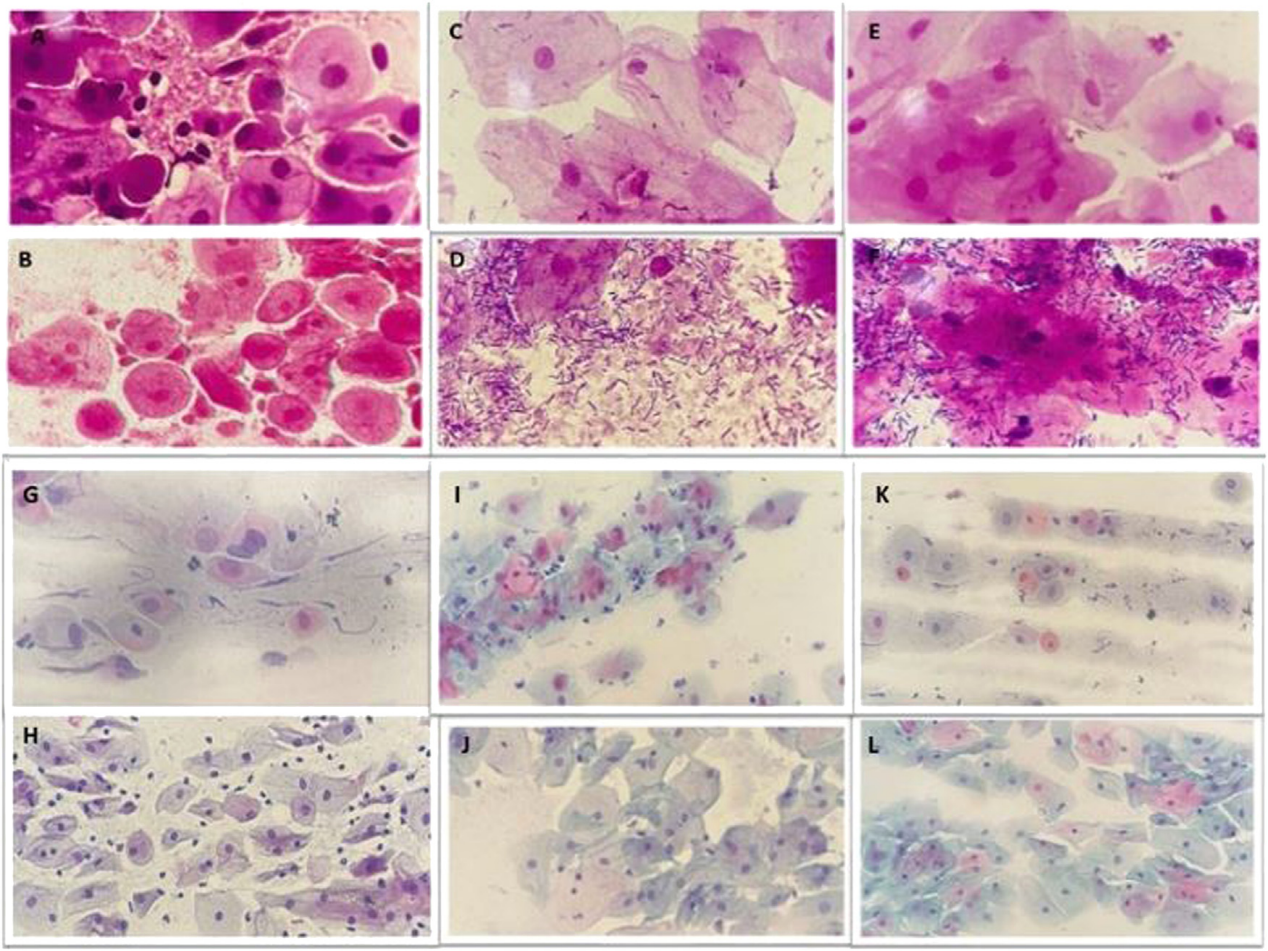
Microscopic observation of the vaginal microbiota (Gram stain 400 to 1000×) and maturation of the vaginal epithelium (Papanicolaou 400×). Vaginal microbiota in the control group: (A) day 0, (B) day 90; in the vaginal estrogen group: (C) day 0, (D) days 90; and in the MAFRF group: (E) day 0, (F) day 90. Vaginal epithelium maturation in the control group: (G) group day 0, (H) day 90; in the vaginal estrogen group: (I) day 0, (J) day 90; and in the MAFRF group: (K) day 0, (L) day 90.

There was an increase in MV scores for women in the MAFRF group of 2.3 points between baseline (50.0) and after 90 days (52.3), *z* = -3.434, p = 0.001, *r* = -0.54, indicating a moderate effect. In the vaginal estrogen group, a significant increase was also observed in the MV scores of 5.93 (95% CI 3.67 to 8.18), *t*(39) = 5.326, p < 0.01, *d* = 0.84 (Fig. 2). There were significant differences between the three groups in the MV score after 90 days (p < 0.01). Post hoc analysis revealed differences in median scores between MAFRF (52.25) and control (48.00) groups (p < 0.01), between vaginal estrogen (53.00) and control (48.00) groups (p < 0.01) and between the MAFRF and vaginal estrogen groups (p = 0.028). In the microscopic observation is possible to see this change of the vaginal microbiota (Gram stain 400 to 1000×) and maturation of the vaginal epithelium. In the microscopic observation is possible to see this change in the vaginal microbiota and maturation of the vaginal epithelium (Fig. 3)

### Adverse events

The patients recovered quickly, and the aftereffects of micro-ablation (burning, redness, irritation of the introitus, vaginal pain) disappeared 3–5 days after the application. Concerning the local side effects, one woman reported burning and redness that lasted 2 to 3 days. None of the patients discontinued the treatment because of the occurrence of the adverse events. Few adverse effects were observed in the E2 group. Five patients complained of vaginal discharge.

## Discussion

In the present study, treatment of vulvovaginal atrophy with MAFRF or with local estrogen therapy yielded equivalent results, and both showed significant improvements over values present in untreated controls. Analysis of the FSFI testing showed an improvement in the “desire domain” for the MAFRF and vaginal estrogen groups compared to pretreatment values and scores in the control group. A pioneering study by Kamilos et al., using the MAFRF, observed an improvement not only in the desired domain but also in the domains of excitement, lubrication, satisfaction, pain, and total score.^7^ Recent studies also demonstrated improvements in sexual function using MAFRF.^7^, ^8^, ^9^ Slongo et al. observed that the total FSFI score improved in the Radiofrequency (RF) group and increased the occurrence of orgasm and desire.^9^ In contrast, recent research using the CO_2_ LASER showed no improvement in FSFI scores when compared to a sham control and vaginal estrogen.^19^^,^^20^ Systematic reviews performed on this topic concluded that despite the improvement in FSFI observed in individual studies, limitations in their designs downgraded the quality of evidence of these findings.^5^^,^^6^^,^^21^

Assessing overall sexual function and satisfaction is difficult as numerous psychological and social factors influence and impair the outcome. In addition, the possible reluctance of subjects to truthfully answer questions of a sexual nature may have also compromised the accuracy of the findings.^1^^,^^22^ Also, other effects may be related to dyspareunia, such as vulvar lichen sclerosus. A previous study has shown that RF could effectively relieve anogenital lichen sclerosus symptoms, especially pruritus, burning sensation, dryness, and dyspareunia.^23^

About the VHI, the authors found improvement in vaginal humidity, fluid volume, pH, elasticity, integrity, and total domain following the MAFRF treatment, as observed in the vaginal estrogen group. The increase in all VHI domains was consistent with findings from a pilot study conducted using the same technique on a comparable population.^8^ According to Slongo et al. comparing the RF with Pelvic Floor Muscle Training (PFMT), the total post-treatment VHI scores for the RF showed superior improvement over that of a PFMT treatment group, and the analyses of vaginal moisture, fluid volume, vaginal pH, integrity and elasticity showed improvement only in the RF.^10^ Leibaschoff et al., using transcutaneous temperature-controlled radiofrequency, also reported VHI improvement.^24^

The present results also demonstrated a return of the vaginal microbiota to *Lactobacillus* dominance after the MAFRF sessions, like that observed in the vaginal estrogen group. Despite the importance of microbiota for vaginal health, few studies have evaluated this parameter. A pilot study performed with the same MAFRF technique reported a significant increase in the number of *Lactobacillus spp*. after three sessions.^8^

Regarding vaginal epithelial maturation, the present results showed an increase in VMI, similar in degree to that of estrogen treatment. In contrast, Slongo et al., observed there was no difference in VMI after treatment with RF.^10^

The main strength of the study is that it is the first randomized clinical trial that evaluates the use of radiofrequency for the management of GSM symptoms. Including, not only subjective outcomes but also objective outcomes (Nugent score and MV). Limitations of the present study include the short follow-up time. It will be necessary to verify the long-term sustainability of the authors’ observations. A higher number of participants is also required to give robustness to the results. Difficulty in patient acceptance of questionnaires (especially regarding sexual-related functions), the use of individual-dependent indices (VHI and VM), and a subjective index (VHI) also can be cited as limitations.

## Conclusion

The radiofrequency treatment is well tolerated and may be an alternative in managing vulvovaginal atrophy. However, additional randomized clinical trials of rigorous methodological quality, ample follow-up time, and a high number of participants are necessary before MAFRAF can be recommended in clinical practice.

## Authors' contributions

Sarmento ACA was responsible for the study conception and design, acquisition of data, analysis, and interpretation of data, drafting of manuscript, and critical revision. Fernandes FS, Maia RR, and Camaro JDAS were responsible for the interpretation of data, drafting of manuscript, and critical revision. Crispim JCO and Júnior JE were responsible for the study conception and design, drafting of the manuscript and critical revision. Gonçalves AK was responsible for the study conception and design, analysis, and interpretation of data, drafting of manuscript and critical revision.

## Funding

This research did not receive any specific grant from funding agencies in the public, commercial, or not-for-profit sectors.

## Declaration of Competing Interest

The authors declare no conflicts of interest.

## Appendix Supplementary materials

Image, application 1
