# Supplementary material for: Microablative fractional radiofrequency for sexual dysfunction and vaginal Trophism: A randomized clinical trial
Source: Clinics (Sao Paulo). 2023 Oct 13;78:100293. doi: 10.1016/j.clinsp.2023.100293 (PMC10589764; doi:10.1016/j.clinsp.2023.100293)
Supplement: Supplementary file 1 [file mmc1.docx]

**Microablative Fractional Radiofrequency for Sexual Dysfunction and Vaginal Tropism: A Randomized Clinical Trial**

Ayane Cristine Alves Sarmento^1,2^ ID:https://orcid.org/0000-0001-9131-1952, Fabíola Sephora Fernandes^2^ ID:https://orcid.org/0000-0002-5661-016, Rafaella Rêgo Maia^1^ ID: https://orcid.org/0000-0001-7819-0690, Juliana Dantas de Araújo Santos Camargo^1^ ID: https://orcid.org/[0000-0001-8692-5706](https://orcid.org/0000-0001-8692-5706), Janaina Cristiana de Oliveira Crispim^2^ ID:https://orcid.org/0000-0002-1344-0078, José Eleutério Júnior^3^ ID: https://orcid.org/0000-0003-4617-726, Ana Kataherine Gonçalves^1,4^* ID:https://orcid.org/0000-0002-8351-5119

^1^Health Sciences Postgraduate Program, Federal University of Rio Grande do Norte (UFRN), Natal, RN, Brazil.

^2^Department of Clinical Analysis and Toxicology, Federal University of Rio Grande do Norte, Natal, Brazil.

^3^Departamento Obstetrics and Gynaecology, Federal University of Ceara, Ceara, CE, Brazil.

^4^Department of Obstetrics and Gynaecology, Federal University of Rio Grande do Norte (UFRN), Natal, RN, Brazil.

*Correspondence

*Correspondence

Ana Katherine Gonçalves, Major Laurentino de Morais St 1218/1301, Natal, RN, Brazil. Email: anakatherine_ufrnet@yahoo.com.br

Gonçalves, AK. ORCID ID: <https://orcid.org/0000-0002-8351-5119>

**AUTHOR CONTRIBUTIONS**

# Sarmento ACA was responsible for the study conception and design, acquisition of data, analysis and interpretation of data, drafting of manuscript and critical revision. Fernandes FS, Maia RR, and Camaro JDAS were responsible for interpretation of data, drafting of manuscript and critical revision. Crispim JCO and Júnior JE were responsible for the study conception and design, drafting of manuscript and critical revision. Gonçalves AK was responsible for the study conception and design, analysis and interpretation of data, drafting of manuscript and critical revision.
